# Optimizing Low-Cost Genotyping and Imputation Strategies for Genomic Selection in Atlantic Salmon

**DOI:** 10.1534/g3.119.400800

**Published:** 2019-12-11

**Authors:** Smaragda Tsairidou, Alastair Hamilton, Diego Robledo, James E. Bron, Ross D. Houston

**Affiliations:** * The Roslin Institute and Royal (Dick) School of Veterinary Studies, University of Edinburgh, Edinburgh, EH25 9RG, United Kingdom,; † Hendrix Genetics Aquaculture BV/ Netherlands Villa ’de Körver’, Spoorstraat 695831 CK Boxmeer, The Netherlands, and; ‡ Institute of Aquaculture, University of Stirling, FK9 4LA, United Kingdom

**Keywords:** Salmon breeding, genotype imputation, aquaculture, sea lice resistance, genomic prediction, Genomic Prediction, GenPred, Shared Data Resources

## Abstract

Genomic selection enables cumulative genetic gains in key production traits such as disease resistance, playing an important role in the economic and environmental sustainability of aquaculture production. However, it requires genome-wide genetic marker data on large populations, which can be prohibitively expensive. Genotype imputation is a cost-effective method for obtaining high-density genotypes, but its value in aquaculture breeding programs which are characterized by large full-sibling families has yet to be fully assessed. The aim of this study was to optimize the use of low-density genotypes and evaluate genotype imputation strategies for cost-effective genomic prediction. Phenotypes and genotypes (78,362 SNPs) were obtained for 610 individuals from a Scottish Atlantic salmon breeding program population (Landcatch, UK) challenged with sea lice, *Lepeophtheirus salmonis*. The genomic prediction accuracy of genomic selection was calculated using GBLUP approaches and compared across SNP panels of varying densities and composition, with and without imputation. Imputation was tested when parents were genotyped for the optimal SNP panel, and offspring were genotyped for a range of lower density imputation panels. Reducing SNP density had little impact on prediction accuracy until 5,000 SNPs, below which the accuracy dropped. Imputation accuracy increased with increasing imputation panel density. Genomic prediction accuracy when offspring were genotyped for just 200 SNPs, and parents for 5,000 SNPs, was 0.53. This accuracy was similar to the full high density and optimal density dataset, and markedly higher than using 200 SNPs without imputation. These results suggest that imputation from very low to medium density can be a cost-effective tool for genomic selection in Atlantic salmon breeding programs.

Genomic selection is increasingly applied in aquaculture breeding to expedite genetic gain for key production and disease resistance traits (Zenger *et al.* 2019). Genomic selection exploits both between- and within-family genetic information, and therefore can predict the breeding values of individuals more accurately than using traditional pedigree-based approaches. However, routine and effective application of genomic selection in breeding programs depends on large training datasets of phenotypes and genotypes, which can be expensive. In livestock and plant breeding, genomic information on large populations can be achieved in a very cost-effective manner by means of genotype imputation. Imputation exploits the presence of haplotypes that are shared between related individuals (Li *et al.* 2009), such that high density (HD) imputed genotypes can be obtained from low density (LD) SNP panels. This can facilitate a major reduction in the cost of genotyping, as direct genotyping at HD is required only for a subset of individuals, *i.e.*, the reference panel. However, applications of genotype imputation in aquaculture breeding are at a formative stage, and optimal strategies need to be defined.

In typical aquaculture breeding programs large full-sibling families are available, and performance testing is routinely performed on siblings of selection candidates. Full-siblings share large genomic segments, which results in relatively high genomic prediction accuracy with low density SNP genotypes, when compared to the SNP densities typically required in other farmed animal species (Tsai *et al.* 2016; Vallejo *et al.* 2017; Palaiokostas *et al.* 2018; Robledo *et al.* 2018; Yoshida *et al.* 2018; Yoshida *et al.* 2019). Further, high density SNP arrays have been developed for Atlantic salmon (Houston *et al.* 2014), and a high quality reference genome assembly is available to map those SNPs and determine their order (Lien *et al.*, 2016). Therefore, given the family structure within a typical aquaculture setting, a first step is to identify the minimum SNP panel density required to achieve maximum genomic prediction accuracy. Furthermore, optimizing the composition of these SNP panels needs to be explored to assess impact on the prediction accuracy. Previous genotype imputation studies have highlighted the potential of harnessing medium or high density genotyping of parents and low density genotyping of offspring to reduce costs of genomic prediction (Tsai *et al.* 2017; Yoshida *et al.* 2018; Dufflocq *et al.* 2019). In these studies, the genomic prediction accuracy that could be achieved when using LD genotypes imputed to HD was found to be comparable to the prediction accuracy when using the full HD panel in Atlantic salmon (Tsai *et al.* 2017; Yoshida *et al.* 2018), and in simulated data for rainbow trout (Dufflocq *et al.* 2019). However, a systematic analysis of the optimal combination of low and high density panels, and of the effects of SNP panel composition, *i.e.*, which SNPs are selected for the reduced density panels, has not been yet performed.

One of the most important target traits for genetic improvement in salmon breeding is host resistance to infectious diseases such as sea lice. Sea lice are parasitic marine copepods of the family of Caligidae, endemic in all major salmon-producing countries (*Lepeophtheirus salmonis* in Europe and North America, and *Caligus rogercresseyi* in Chile), and it is the most costly disease-related problem for the global salmon industry (total cost of sea lice control is estimated to be €305 million in 2006 (Costello 2009)). Sea lice infestation has a severe negative impact on salmon health and production, causing substantial financial losses due to treatment costs, associated labor and increased morbidity and mortality due to infestation or secondary bacterial and fungal infections (Gjerde *et al.* 2011; Bruno *et al.* 2013). In addition, sea lice show variability in their sensitivity to common treatments, and in some instances, they express high levels of resistance to commonly-used therapeutants (Tully and Mcfadden 2000; Sevatdal and Horsberg 2003). Encouragingly, host resistance to sea lice has shown significant heritabilities of 0.22-0.33 (*e.g.*, Gjerde *et al.* 2011; Tsai *et al.* 2016). Another economically important production trait is body weight, which reflects growth performance. Body weight has been reported to have a high heritability of 0.5-0.6 (*e.g.*, Tsai *et al.* 2015). In addition, both sea lice resistance and body weight are known to have a polygenic genetic architecture (Tsai *et al.* 2015; Tsai *et al.* 2016). Hence, these traits lend themselves to improvement by genomic selection approaches, and cost-efficient genotyping approaches employing genotype imputation may encourage widespread commercial adoption (Tsai *et al.* 2017).

The aim of this study was to systematically evaluate and develop optimal genotype imputation strategies for Atlantic salmon breeding programs. Using data from a typical commercial breeding program, several genotype imputation strategies for genomic selection were tested by (a) identifying high density SNP panels with optimal density and composition; (b) testing the imputation accuracy when parents were genotyped at high density, and offspring at a range of low densities (c) comparing the genomic prediction accuracies of each of the datasets, and (d) assessing the cost-benefit of imputation for genomic prediction by quantifying the trade-off between the cost of genotyping at a given density and the achieved improvement in genomic prediction accuracy.

## Materials and Methods

### Phenotypes and data transformations

The data used for testing genotype imputation and genomic prediction were collected from an Atlantic salmon commercial population (Landcatch, UK), as described in (Tsai *et al.* 2015). Briefly, the data comprised of 520 phenotyped and genotyped post-smolt salmon (267 males and 253 females), offspring of 29 sires and 57 dams. Each sire was mated to two dams (except for one sire mated to one dam), so that the offspring were full-sibs / paternal half-sibs, derived from 57 nuclear families with 4 - 14 offspring per family.

The population was challenged with sea lice (*Lepeophtheirus salmonis*) as part of a trial conducted at the Marine Environmental Research Laboratory (Machrihanish, UK) in 2007, as described in (Tsai *et al.* 2015). Phenotypic records of sea lice counts (SLC) were collected for all offspring. SLC ranged from 1 to 81 lice per animal, and had a positively skewed distribution with a mean of 25.5 and median 23.5 (Supplementary Information 1). SLC was logarithmically transformed and log_e_(SLC), with a mean of 3.11 and median of 3.16, was used in all subsequent analysis (Supplementary Information 1). The weight of the offspring ranged from 52 to 203 g with a mean of 111.8 g. The weight data were normally distributed and therefore no transformation was required.

### Genotypes and quality control

All samples were genotyped with the Affymetrix Axiom 132K Atlantic salmon SNP chip (Houston *et al.* 2014) such that genotypes were available for all animals for 78,362 high quality, mapped and ordered SNP markers. Families with > 5% and individuals with > 10% Mendelian errors were excluded using Plink/1.90-beta4.1 (Purcell *et al.* 2007). Further quality control was conducted for the offspring using the GenABEL package (Aulchenko *et al.* 2007) on R/3.1.2 (R Development Core Team 2008) with the following criteria: SNPs with a minor allele frequency (MAF) < 5%, call rates < 95%, or Hardy-Weinberg Equilibrium P-value < 10^−5^ were excluded. Individuals with a percentage of missing genotypes > 3% were also excluded. In total, 76,488 SNPs (hereafter referred to as the HD SNP panel), and 520 offspring passed all criteria, and were used to calculate the genomic relationship matrices for each SNP panel.

### Calculating genomic relationships

The identity by state (IBS) genomic relationship matrix (**G**) was calculated from the GenABEL/R (“ibs” function) kinship matrix (Amin *et al.* 2007) multiplied by two, so that the genomic relationship between animals *i* and *j* was given by:

$$g_{ij}=\frac{1}{n}\sum_{k=1}^{n}\frac{\left(x_{ik}-2p_{k})(x_{jk}-2p_{k}\right)}{2p_{k}(1-p_{k})}$$

Where *n* is the number of loci used for estimating relationships; *x_ik_* is the count of alternative alleles (0, 1 or 2) of individual *i* at SNP locus *k* where the reference allele is arbitrarily chosen; and, *p_k_* is the frequency of the reference allele in the data.

The diagonal elements were given by *g_ii_ = 1+F_i_*, where F_i_ is the inbreeding coefficient for individual *i*, calculated using SNP genotypes as follows: $F_{i}=n^{-1}\sum_{k=1}^{n}\frac{\left(H_{E,k}-H_{ik}\right)}{H_{E,k}}$, where *H_E,k_* is the expected heterozygosity at locus *k* assuming Hardy‐Weinberg equilibrium (HWE); and *H_ik_* is the observed heterozygosity for animal *i*. Subsequently **G** was inverted (R “solve” function) to be used in the ASReml (Gilmour *et al.* 2009) analysis described below.

Structure exploration using classical multi-dimensional scaling analysis (function “cmdscale” in package “stats”/R/3.1.2) did not identify any sub-clustering in the data (Supplementary Information 1).

### Statistical Analysis

The SNP panels used throughout the analysis were defined as follows: ‘*HD SNP panel*’ was the full high density panel containing 76,488 SNPs after quality control; ‘*reduced SNP panels*’ were the reduced density panels generated from the HD SNP panel to test genomic prediction at lower SNP densities; ‘*optimal SNP panel*’ (medium density) was the selected 5K SNP panel (see ‘Identification of optimal SNP panels’ section); and, ‘*imputation SNP panels*’ were the low density SNP panels assumed to be genotyped on the offspring, selected from the optimal SNP panel to test imputation.

The analyses comprised three main parts: (a) identification of optimal SNP panels in terms of density and SNP composition; (b) assessment of the imputation accuracy of a range of lower densities of imputation SNP panels, when imputed to the optimal SNP panel identified in (a); and, (c) estimation of genomic prediction accuracies of imputed datasets and comparison with genomic prediction accuracies of datasets without imputation. All analyses were performed via a custom pipeline that was developed integrating: (i) PLINK /1.90-beta4.1 (Purcell *et al.* 2007), R/3.1.2 and Shell for selection of SNP panels, and calculation of genomic relationship matrices; (ii) FImpute/2.2 (Sargolzaei *et al.* 2014) for genotype imputation; and (iii) ASReml/3.0 for estimating breeding values. The code used for the construction of SNP panels and for the multiple cross-validation procedure can be accessed on GitHub (https://github.com/SmaragdaT/CVrep) and is available as an R package (CVrepGPAcalc v1.0 / R version 3.1.2) under GNU General Public License v3.0 (Tsairidou 2019; CVrepGPAcalc v1.0).

#### Identification of optimal SNP panels:

Previous research has shown that genomic prediction accuracy has little or no reduction as SNP panel density is reduced from 132K down to approximately 5K (Tsai *et al.* 2016). The focus of the selection of optimal SNP panels was on identifying an approximate minimal SNP density which could be considered the asymptote of prediction accuracy. From the HD SNP panel SNP, reduced panels were generated for densities of 60,000, 10,000, 5,000, 2,000, and from 1,000 down to 200 SNPs at 100 SNP density intervals, using in-house built software which is available as an R/3.1.2 package (Tsairidou 2019; CVrepGPAcalc v1.0). Two different methods for selecting SNPs were used: (i) SNPs were sampled randomly without replacement across the entire genome; and (ii) SNPs were sampled randomly, without replacement, within each chromosome, and the number of SNPs sampled from each chromosome was proportional to the physical length of the chromosome as calculated using the *salmo salar* reference genome assembly (Lien *et al.* 2016 Genbank accession GCA_000233375.4). For method (ii), in some instances, the total number of SNPs selected across the genome was allowed to be slightly higher than the target density due to rounding. Furthermore, for higher densities, the number of SNPs available on some chromosomes could be smaller than the required number, therefore the total number of SNPs selected across the genome was allowed to be smaller than the intended density (Supplementary Information 2; Table S1).

**Figure 1:**
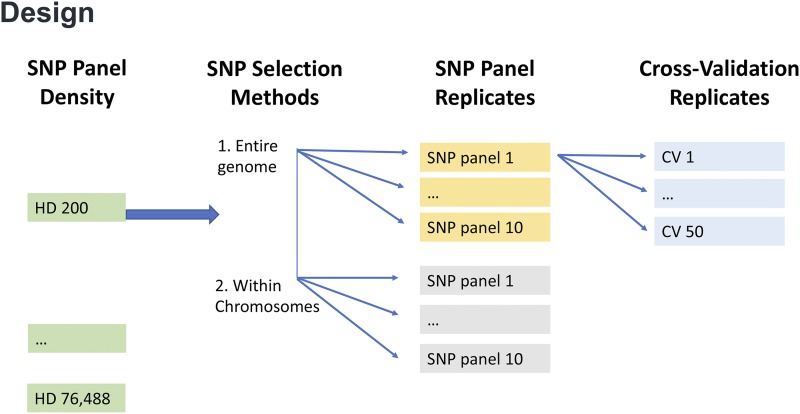
Experimental design.

The process was replicated 10 times so that for each density, 10 SNP panels were generated, which were allowed to partly overlap by chance (Figure 1). For each density and each replicate, the genomic relationship matrices were calculated for the offspring as described above using the SNPs that passed quality control.

An additional method for SNP selection was trialled whereby SNPs were chosen based on even physical distance intervals, but results were similar to the two random selection methods described above, and are reported in Supplementary Information 3 (Figure S3). Briefly, SNPs were selected so that they would be on average equally spaced within each chromosome with a ‘step’ of 0.5, 1, 3, 5, 7, 9, and 11 Mbp between them. The first and last SNPs on each chromosome were always selected. If given the ‘step’ and physical position of the subsequent SNP, the same SNP happened to be selected twice, then the next SNP was included in the panel instead. The larger the ‘step’ the lower the density (Supplementary Information 3; Table S3).

##### Cross validation to test genomic prediction accuracy

Genomic prediction accuracy in the offspring was assessed through cross validation. The offspring were randomly partitioned into five groups of equal size, each time masking the phenotypes of one of the groups (validation set, n_v_ = 104). Subsequently, using the phenotypes of the remaining four groups (training set n_t_ = 416) and the genomic relationship matrix, breeding values (BVs) were predicted for each of the validation sets fitting the following mixed model in ASReml/3.0:

y=m1+Xb+Za+e (1)

Where, **y** is the response variable (Log_e_(SLC) or body weight); **m** is the overall mean, **b** is the vector of fixed effects (sex and weight for the Log_e_(SLC) model; sex for the body weight model) associated with the incidence matrix **X**; **a** is the vector of random animal effects with **a**∼**MVN**(0,**G**σ_a_^2^) associated with the incidence matrix **Z,** where σ_a_^2^ is the additive genetic variance; and, **e** is the residual error with **e**∼**MVN**(0,**I**σ_e_^2^). The mean correlation between the predicted BVs (*ŷ*) and the phenotypes (*y*) was calculated for the five validation sets.

In order to assess the effects of random sampling on the prediction accuracy (Tsairidou *et al.* 2014), the cross-validation process was replicated fifty times, where for each replicate a new randomization of individuals into groups was performed. For each high density SNP panel, the overall mean correlation and standard deviation were obtained over the 50 replicates (Figure 1).

For each SNP panel and for each replicate, prediction accuracy was calculated as the mean correlation divided by the square root of the heritability $\approx r(y,\hat{y})/h$, for Log_e_(SLC) and for weight. This heritability was calculated from the whole dataset, all 520 offspring and with **G** calculated on all SNP markers after quality control with the same criteria as above (76,488 SNPs), and using model (1). Then for each SNP density, the average accuracy (and standard deviation) was obtained over the 10 SNP panel replicates.

#### Imputation SNP panels and assessment of imputation accuracy:

A range of imputation SNP panels were constructed as a subset of the optimal SNP panel identified in (a) (*i.e.*, the lowest density SNP panel that rendered genomic prediction accuracies for both traits similar to those obtained with the HD SNP panel). For the imputation panels, SNPs were selected randomly within each chromosome (proportional to chromosome length as previously described). Due to rounding, it was possible that the total number of selected SNPs could be slightly larger than the intended density (Supplementary Information 2; Table S2). The optimal SNP panel was assumed to be genotyped on all parents (n = 86) and the imputation SNP panels of varying densities were assumed to be genotyped for all offspring (n = 520), so that the offspring were imputed to the optimal SNP panel density (parents’ genotypes). Imputation was conducted using FImpute/2.2 (Sargolzaei *et al.* 2014). Imputation accuracy was calculated as the correlation between imputed and observed genotypes in the offspring (Yoshida *et al.* 2018). Then the average imputation accuracy across individuals was calculated for each of the low density imputed SNP panels.

#### Genomic prediction accuracies of imputed datasets:

Estimated breeding values for log_e_(SLC) and body weight, and cross-validated prediction accuracies were estimated in ASReml/3.0 using model (1), with the difference that here the **G** matrices were calculated from both directly genotyped and imputed SNP genotypes for the offspring. Quality control analysis described in section ‘Genotypes and quality control’ was repeated for the imputed genotypes. The multiple cross-validation procedure was used as described above, and for each imputed dataset, the average prediction accuracy was calculated over 50 replicates.

### Cost-benefit analysis using imputed data

A cost-benefit analysis was performed to evaluate the economic benefit of genotyping the selection candidates at lower densities and performing genotype imputation considering the genomic prediction accuracy obtained. The scenarios tested were (i) all animals (parents and offspring) genotyped at medium density with a 5K SNP panel, and, (ii) parents genotyped at 5K and offspring at 200 SNPs and imputed to 5K. The annual cost of genotyping was estimated as follows: for a salmon breeding program assuming 300 parents and 10,000 offspring, for scenario (i) a cost of $15 per individual was assumed; for scenario (ii) 300 parents genotyped at 5K were assumed to cost $30 per SNP array (the price per array increases with lower volume of samples), and 10,000 offspring genotyped at 200 SNPs were assumed to cost $5 per individual to genotype the SNP panel.

### Data availability

The code used in the current study can be accessed on GitHub (https://github.com/SmaragdaT/CVrep), and is available as an R package (CVrepGPAcalc v1.0 / R version 3.1.2) under GNU General Public License v3.0 (Tsairidou 2019; CVrepGPAcalc v1.0). To install in RStudio use: install.packages(“devtools”); library(devtools); install_github(“SmaragdaT/CVrep”, subdir=”CVrepGPAcalc”); library(CVrepGPAcalc). The genotype and phenotype data can be accessed at https://www.g3journal.org/content/7/4/1377.supplemental (Tsai *et al.* 2017). Supplementary Information 1: sea lice count distribution and data structure exploration using classical multi-dimensional scaling; Supplementary Information 2: number of SNPs selected when SNPs were sampled randomly within each chromosome; Supplementary Information 3: genomic prediction accuracies when SNPs were selected based on physical distance; Supplementary Information 4: correlations between phenotypes and cross-validated predicted BVs for the different HD SNP panel densities; Supplementary Information 5: distributions of imputation accuracy values across individual animals; Supplementary Information 6: genomic prediction accuracy after removing SNPs with minor allele frequency less than 0.3. Supplemental material available at figshare: https://doi.org/10.25387/g3.9929945.

## Results

### Genetic parameters and testing of genomic prediction with reduced density panels

The genomic heritability using the HD SNP panel was found to be 0.19 (SE = 0.07) for sea lice resistance, and 0.57 (SE = 0.07) for body weight which was generally in agreement with findings from earlier studies, either based on the same (*e.g.*, Tsai *et al.* 2015; Tsai *et al.* 2016) or different populations (Cáceres *et al.* 2019).

**Figure 2:**
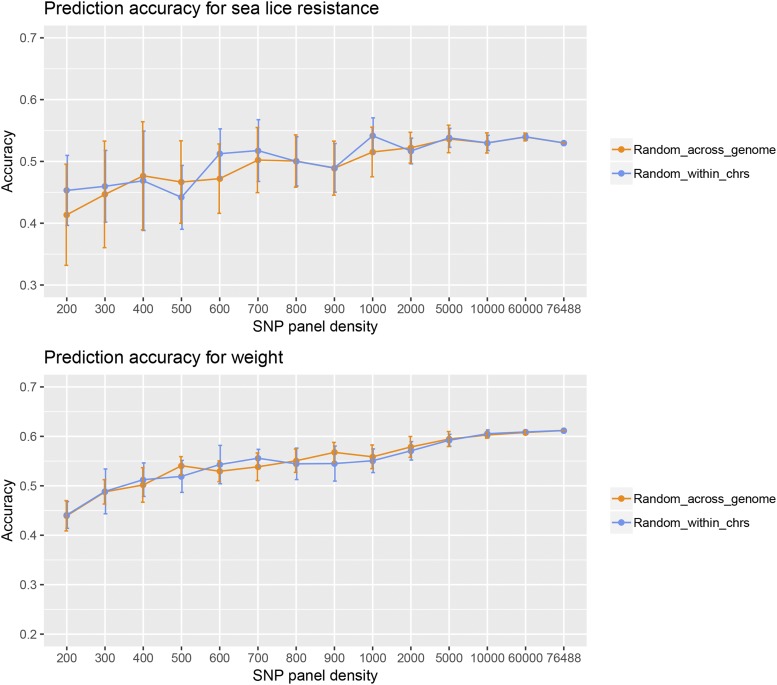
Prediction accuracy for sea lice resistance and body weight for the HD and the reduced SNP panels, when SNPs were sampled (i) randomly across the genome, and (ii) randomly within each chromosome, proportionally to chromosome length. Vertical bars represent the standard deviations over 10 SNP panel replicates.

For the different reduced SNP panel densities the correlations between phenotypes and cross-validated predicted BVs reflected the increase in information with increasing SNP density (Supplementary Information 4). Reducing the SNP panel density from the HD SNP panel (76,488 SNPs) to 200 SNPs resulted in a 14.5% decrease in genomic prediction accuracy for sea lice resistance, and a 27.9% decrease for body weight (Figure 2; SNPs selected randomly within chromosome). Across all SNP densities, prediction accuracies for body weight were higher than those for sea lice resistance. Predictions for weight appeared to benefit slightly more from using higher SNP densities compared to sea lice resistance (Figure 2), which may reflect that body weight is potentially a more polygenic trait than sea lice resistance. Both methods of selecting SNPs to construct reduced SNP panels (randomly across the entire genome or within each chromosome) showed similar patterns performed similarly with regards to genomic prediction accuracy.

Regardless of the trait, prediction accuracy started to decrease between 2,000 and 5,000 SNPs, therefore the medium SNP density of 5,000 was tested as the optimal SNP panel for the imputation analyses. This medium SNP density was used rather than the HD SNP panel because it was assumed that a 5K SNP panel would be cheaper to genotype in most circumstances.

### Variability of cross-validated prediction accuracy

**Figure 3:**
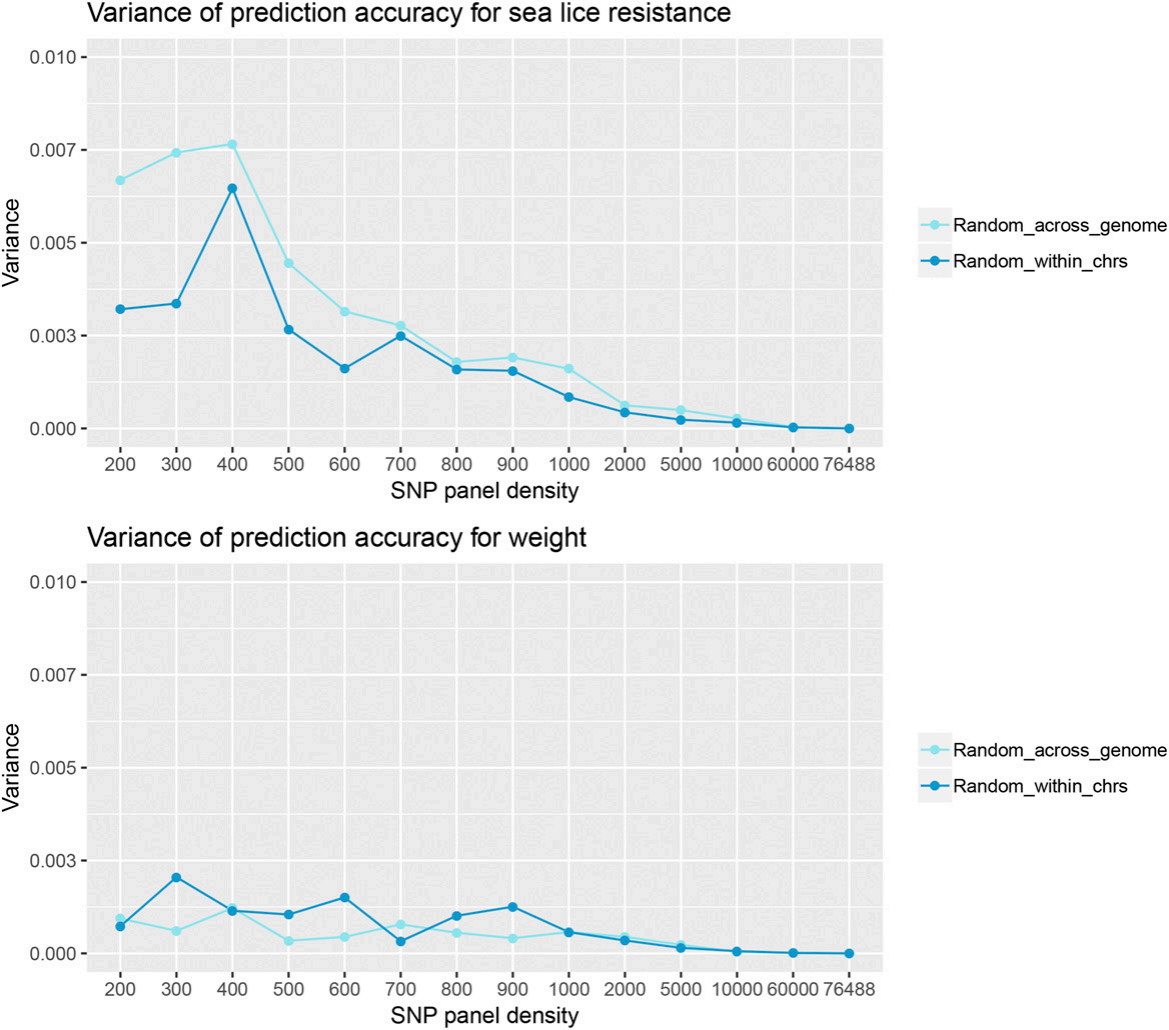
Variance of prediction accuracy of the reduced SNP panels, for sea lice resistance and weight, when SNPs were sampled (i) randomly across the genome, and (ii) randomly within each chromosome, proportionally to chromosome length.

While the reduction in genomic prediction accuracy with the reduced SNP densities was modest, the standard deviations (Figure 2) and the variances (Figure 3) were substantially larger at these lower densities. In other words, there was substantial variability in accuracy between SNP panel replicates at lower densities, which means the accuracy depends on the SNPs included on the panel. In contrast, variability over SNP panel replicates diminished for SNP densities of 5K of larger. This phenomenon was more pronounced for sea lice resistance than for body weight (Figure 3).

**Figure 4:**
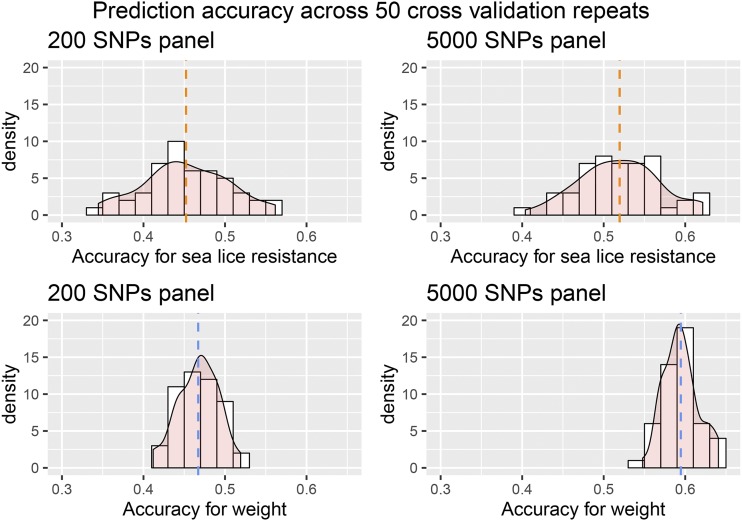
Prediction accuracy across 50 cross-validation repeats for the 200 and 5000 SNP panels, for sea lice resistance and body weight.

Another source of variation in the genomic prediction accuracy is due to the random sampling of individuals into the cross-validation groups, which will depend to some extent on sample size. Interestingly, a single prediction accuracy from one cross-validation can be a major over- or underestimate of the prediction accuracy. For example, for one of the medium density 5000 SNP panels, the prediction accuracy for sea lice resistance ranged from 0.40 to 0.62 over the 50 cross-validation replicates, with a mean of 0.52 (Figure 4). Similarly for weight and for the same 5000 SNP panel, the prediction accuracy ranged from 0.55 to 0.64, with a mean of 0.59 (Figure 4). Therefore, in the present study a multiple cross-validation procedure was followed, where individuals were re-allocated into 5 cross-validation groups 50 times. A larger sampling size would be expected to make the distribution of the prediction accuracies narrower and minimize the random sampling effects. This may be good practice for future studies of genomic selection in aquaculture breeding to avoid sampling bias.

### Imputation accuracy

**Figure 5:**
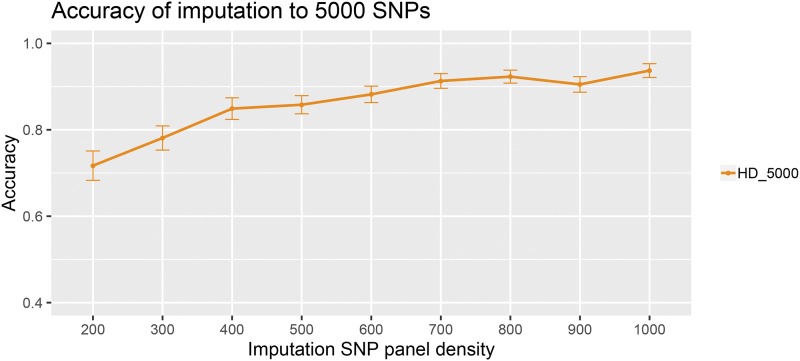
Imputation accuracy for the imputation SNP panels, when imputed to the optimal SNP panel. Vertical bars represent the standard deviations over 520 offspring.

Imputation accuracy increased as the density of the imputation SNP panels increased (Figure 5) which was as expected based on previous studies (*e.g.*, Hayes *et al.* 2012). For imputation to 5000 SNPs, the accuracy ranged from 0.72 (SD = 0.03) for 200 SNPs, to 0.94 (SD = 0.02) for the SNP panel comprising 1000 SNPs, reaching > 90% imputation accuracy for 700 SNPs or more. The standard deviation of the imputation accuracies among individuals reduced for higher density imputation panels (SD = 0.034 for 200 SNPs, and SD = 0.016 for 1000 SNPs; Figure 5). Imputation accuracy per individual ranged from 0.61 to 0.82 when imputing from 200 SNPs, and from 0.86 to 0.96 when imputing from 1000 SNPs (Supplementary Information 5).

### Genomic prediction accuracy of imputed datasets

**Figure 6:**
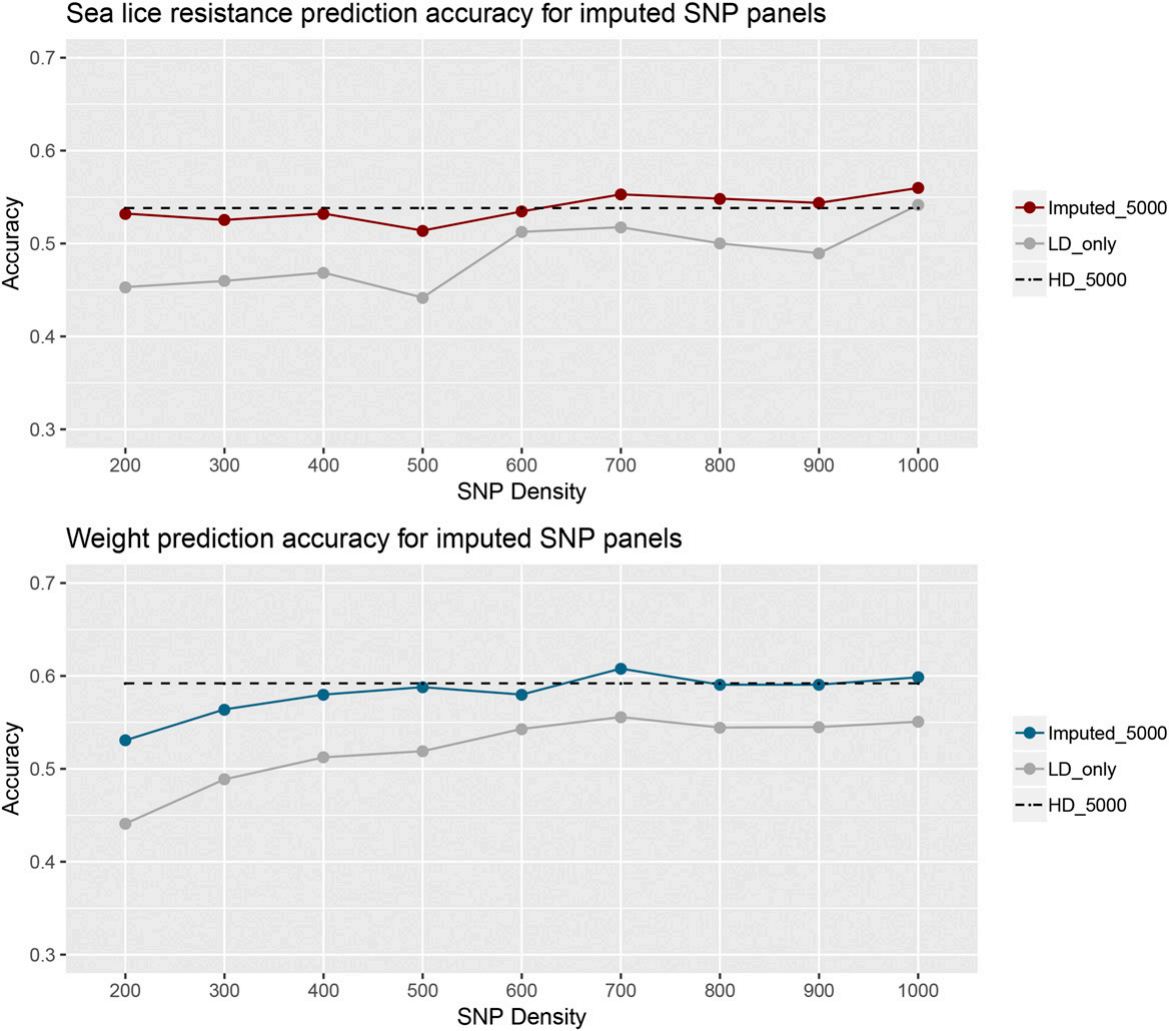
Prediction accuracy of imputation SNP panels for sea lice resistance and body weight, for (i) the imputation SNP panel densities with range from 200 to 1,000 SNPs imputed to the optimal 5,000 SNP panel (Imputed_5000), (ii) the imputation SNP panel densities with range from 200 to 1,000 SNPs alone without imputation (LD_only), and, (iii) the optimal 5,000 SNP panel directly genotyped without imputation (HD_5000).

Despite the higher imputation accuracy of higher density imputation SNP panels (Figure 5), increasing the density of the imputation SNP panel for sea lice resistance resulted in an only marginal increase in genomic prediction accuracy achieved using imputed genotypes (Figure 6). For example, 200 SNPs imputed to 5,000 provided a prediction accuracy of 0.53, while the prediction accuracy for 1000 SNPs imputed to 5,000 was 0.56, and with 5,000 true genotypes was 0.54. For body weight, genomic prediction accuracies using SNPs imputed to 5,000 were slightly higher when higher imputation SNP panel densities were used (Figure 6).

While the influence of the SNP density of the imputation panel on genomic prediction accuracy might be minor, the benefit of imputation *vs.* using the low-density SNP panel alone (without imputation) varied according to SNP density. As a general pattern, genomic prediction accuracies using genotypes imputed to the medium density 5,000 SNPs optimal panel were very close to those using true genotypes for the same density (Figures 2 and 6). The benefits of imputation were highest at the lowest imputation SNP panel densities. For example, genomic prediction accuracy using 200 SNPs without imputation for sea lice resistance was 0.45 (Figure 2 and 6), whereas with imputation to 5,000 SNPs it was 0.53, which corresponds to an increase of 17.5% (Figure 6). In contrast, for a density of 700 SNPs, the benefit of imputation *vs.* no imputation was approximately 6.83%, and at 1,000 SNPs the benefit was negligible. In summary, the use of low density imputation SNP panels (*e.g.*, 200 SNPs) with imputation to medium density (5,000 SNPs) resulted in prediction accuracies comparable to using the optimal SNP panel (5,000 SNPs) on all animals, and significantly outperformed low density panels alone without imputation.

### Cost-benefit analysis

In scenario (i) where all animals (parents and offspring) were genotyped with a medium density SNP panel (5000 SNPs), the total cost of genotyping was estimated at $154,500. However in scenario (ii) where only the parents were genotyped for medium density and the offspring were genotyped for 200 SNPs, the cost was estimated to reduce to $59,000. This corresponds to a 62% cost reduction between scenario (i) and (ii), with virtually no loss of genomic prediction accuracy for the traits measured.

## Discussion

This study explores developing optimal methods for cost-effective genomic selection in aquaculture, with a focus on an Atlantic salmon sib-testing breeding program. In this study, genetic relationships were incorporated through the genomic relationship matrix which was computed from the SNP genotype data and captures identity by state (IBS). While family-based selection using phenotypes and pedigrees can only capture between-family variation, genomic selection using genetic markers can also capture within-family variation. This allows to distinguish between the genetic merit of full-siblings by predicting and utilizing the Mendelian segregation term. Previous studies have shown that using genomic relationships improves the predictive ability of the BVs compared to pedigree-based predictions (Habier *et al.* 2007; Zenger *et al.* 2019). Genomic selection presents further advantages over pedigree-based selection as it allows to reduce emphasis on particular families and therefore facilitates improved control of the rate of inbreeding (Daetwyler *et al.* 2008).

### Genomic prediction using low density SNP panels

The results presented herein confirmed findings from earlier studies of genomic selection in aquaculture species where relatively low density SNP panels were found to be as effective as full high density panels for the prediction of breeding values (Tsai *et al.* 2016; Vallejo *et al.* 2017; Palaiokostas *et al.* 2018; Robledo *et al.* 2018; Yoshida *et al.* 2018; Yoshida *et al.* 2019). Aquaculture settings are typically characterized by full-sib testing schemes, and full-siblings share long genomic segments which can be adequately captured by fewer SNPs. Therefore, the existence of close relationships between training and validation animals is likely to be the reason why fewer SNPs are sufficient to capture relationships between individuals and provide near-maximal prediction accuracy. However, the current study has highlighted high variability in prediction accuracies with lower density SNP panels, reflecting the importance of the sampling effect in selecting the SNP panel. In contrast, for densities > 5K, the risk of losing accuracy due to the chance inclusion of less informative SNP markers diminished. Non-random SNP selection, based on knowledge of the causal mutations affecting the trait of interest, is expected to enhance genomic prediction accuracy and assist particularly with cross-population prediction (Edwards *et al.*, 2016). Such knowledge will allow including the causative variant itself rather than relying on linkage disequilibrium between the QTL and the SNP marker, and prioritizing functional SNPs into genomic prediction.

It should be noted that the high prediction accuracies using relatively modest SNP marker densities is likely to be restricted to sibling-testing schemes or similar. In situations where the relationship between the training and validation populations is more distant (such as use of one population as training set and another population as validation set), prediction accuracy is likely to be much lower, and higher SNP marker density may be more advantageous (Tsai *et al.*, 2016). This is because variation in linkage disequilibrium patterns between SNP markers and QTL across populations compromises across-population prediction accuracy (Snelling *et al.* 2013). Therefore, the results in the present study may be to some extent population and trait specific. Another factor that may affect the prediction accuracy is the minor allele frequency, however, rarer alleles may be important for capturing haplotypes for specific families. Removing SNPs with minor allele frequency less than 0.3 was not found to reduce the variability of prediction accuracy values obtained across the SNP panel replicates (Supplementary Information 6).

Selecting SNPs randomly across the genome or randomly within each chromosome performed similarly in terms of genomic prediction accuracy. The method of random sampling within each chromosome was preferred for the selection of the optimal SNP panel, as this method ensures a more even distribution of SNPs across the genome, and ensures that SNPs from all chromosomes are included in the calculation of the genomic relationship matrices. In the present study, SNPs were selected at random within each chromosome, with the number of SNPs corresponding to the physical length of the chromosome. SNP panels were also constructed by selecting SNPs based on even physical distance within each chromosome (Supplementary Information 3). Selecting SNPs based on genetic distance was not tested, and may be expected to impact on prediction accuracy due to major variation in recombination rate across the genome of salmon, particularly in males. However, given that reducing SNP density from the highest density to the optimal density panel did not reduce prediction accuracy, it is unlikely that genetic distance or regions of high recombination have a major impact.

### Heritability estimation and prediction accuracy

Heritabilities estimated using lower density SNP panels (where the reduced SNP panels were used to calculate the **G** matrix) were not found to be reliable, with large standard deviations that often were larger than the estimates. Hence, while the correlation between predicted BV and phenotype reduced for lower densities as expected, the accuracies calculated using the heritability estimates from lower density SNP panels appeared to remain high (preliminary analysis; results not shown), which was an artifact introduced by the spurious heritability estimates. This is likely to be common in studies of genomic prediction accuracies with reduced marker densities if the reduced panel is used to calculate the h^2^ estimate that underlies the transformation from correlation between phenotypes and EBVs to the accuracy value $r(y,\hat{y})/h$. Therefore, for the calculation of the accuracies presented here, the heritability calculated from the HD SNP panel was used, which is expected to be closer to the true heritability in the population as a greater proportion of the genetic variance is captured by the HD SNP markers. The heritability estimated using the HD SNP panel was consistent with the heritability estimated using pedigree data for the same population (pedigree-based heritability of 0.23 (SE = 0.08) for sea lice resistance).

Genomic prediction accuracy is affected by the genetic distance between training and test populations (*e.g.*, Scutari *et al.* 2016), and the rapid drop in accuracy when the training and test sets do not include close relatives has been observed in both salmon (Tsai *et al.* 2016) and common carp (Palaiokostas *et al.* 2019). The variation observed in the accuracy across the cross-validation repeats is due to the random sampling of individuals to construct the training and validation sets; the genetic distance between the training and validation sets varies by chance and affects the prediction accuracy. Hence in the present study average prediction accuracies are reported over 50 replicates of cross-validation, where in each replicate, individuals are re-allocated in training and test sets. Therefore, where possible in aquaculture breeding programs, close relatives (such as full-siblings) to the selection candidates should be included in reference populations to achieve high accuracy levels for genomic prediction.

### Imputation and cost-benefit analysis

Results from using imputed genotypes for genomic prediction were in agreement with previous studies (Tsai *et al.* 2017; Yoshida *et al.* 2018), where the authors observed that genomic prediction accuracies using imputed genotypes were very close to those of true genotypes. For very low imputation SNP panel densities (*e.g.*, 200 SNPs), although the imputation accuracy was lower, the genomic prediction accuracy using the imputed genotypes was not greatly reduced. The high imputation accuracies observed in this study can be explained by the inclusion of close relatives in the reference group. A trade-off was observed between genotyping cost and loss of prediction accuracy, but using low density imputation panels with imputation to medium density appears to be a highly accurate cost-effective option. A reduction in prediction accuracy means a reduction in genetic gain, and this has a cost implication. However, this cost is difficult to quantify, as it will be specific to the trait, the breeding goal, and the company. Furthermore, it is unclear where this cost would be incurred, for example to the breeding company or to the producer. In addition, it should be noted that this is a small-scale trial and the imputation and genomic prediction performance is likely to be more consistent in larger commercial operations, and therefore needs to be tested for different populations and traits.

## Conclusion

High genotype imputation accuracy can be achieved in the context of a salmon breeding program by genotyping parents at medium density and offspring at low density. When offspring are genotyped at very low density (*e.g.*, 200 SNPs), and the parents are genotyped at medium density (*e.g.*, 5000 SNPs), this is sufficient to obtain near-maximal genomic prediction accuracy. Since genotyping for very low density panels is considerably cheaper than genotyping for medium or high density panels, this genotype imputation approach may be a cost effective tool for genomic prediction in Atlantic salmon.
